# Associations Between Occupational Exposures to Volatile Organic Compounds (VOCs) and Sleep Problems

**DOI:** 10.3389/phrs.2025.1608224

**Published:** 2025-09-25

**Authors:** Amir Hossein Khoshakhlagh, Saeid Yazdanirad, Christopher Drake, Justin Iqal, Dinh Bui

**Affiliations:** ^1^ Social Determinants of Health (SDH) Research Center, Kashan University of Medical Sciences, Kashan, Iran; ^2^ Social Determinants of Health Research Center, Shahrekord University of Medical Sciences, Shahrekord, Iran; ^3^ Henry Ford Health System, Sleep Disorders and Research Center, Detroit, MI, United States; ^4^ Allergy and Lung Health Unit, School of Population and Global Health, The University of Melbourne Australia, Parkville, VIC, Australia

**Keywords:** systematic review, meta-analysis, sleep, occupational exposure, VOCs

## Abstract

**Objectives:**

The purpose of this study, as a systematic review and meta-analysis, is to summarize the evidence on the effects of occupational exposure to VOCs on sleep quality.

**Methods:**

We used five electronic bibliographic databases to identify eligible studies. Three groups of keywords were applied in the search strategy. In evaluating the quality of studies and risk of bias, we utilized the Joanna Briggs Institute tools and the Risk of Bias in Non-randomized Studies of Exposures (ROBINS-E) instruments, respectively. The pooled values were also calculated by meta-analysis.

**Results:**

37 articles were included in the study. There was a consistent finding that exposure to volatile organic compounds (VOCs) adversely affected sleep in workers across various professions. The pooled value of the odds ratio of sleep problems because of exposure to VOCs was 1.60 [95% CI (1.01, 2.19)].

**Conclusion:**

Most studies indicated that occupational exposure to VOCs can significantly influence the sleep of workers in various professions. The exposure can be associated with a variety of sleep problems.

## Introduction

Atmospheric volatile organic compounds (VOCs) are comprised of thousands of gaseous organic trace species [1]. These compounds have a high vapor pressure and low water solubility. Those can be directly emitted from biogenic and anthropogenic sources [1]. Many VOCs are human-made chemicals that are applied in the manufacture of paints, pharmaceuticals, and refrigerants. VOCs are typically used as industrial solvents and those are often components of petroleum fuels, hydraulic fluids, paint thinners, and dry-cleaning agents. VOCs are common ground-water contaminants. Some known VOCs include benzene, toluene, xylene, and ethylbenzene [2].

There are various professions in which workers are exposed to volatile organic compounds (VOCs). For instance, Jo and Song discovered that individuals working in occupations with exposure to gasoline vapor emissions and motor vehicle exhaust significantly experience exposure to elevated levels of aromatic VOCs during their work. Some of these occupations include service station workers, traffic policemen, and parking garage workers, [3]. Previous studies have also identified other occupations at risk of VOC exposure, such as those in the petroleum industry [4], basic iron and steel manufacturing factory [5], automobile-producing factories [6], painting units of automobile-producing factories [7], and gasoline and Compressed Natural Gas (CNG) stations [8]. Furthermore, there are VOCs exposure in small occupations, such as repair shops laundries, and restaurants continuously emit VOCs [9, 10].

Exposure to VOCs may have adverse health consequences including the effects on the nervous system, skin, and respiratory. Moreover, there is emerging evidence that exposure to VOCs may be associated with sleep problems. In the present systematic review and meta-analysis, VOCs were selected to investigate their effect on sleep problems for several reasons. First, exposure to VOCs are currently found in various workplaces, and those are introduced as one of common indoor and outdoor air pollutants in occupational settings [11]. Second, the findings of various studies demonstrated that exposure to VOCs can be associated with serious physiological and psychological effects [12, 13]. As physiological consequences, exposure to VOCs can lead to neurobehavioral consequences, chronic bronchitis, sleep apnea, and allergic reactions [12, 14–16]. In addition to physiological consequences, psychological effects due to exposure to VOCs such as stress and anxiety can be created because of annoyance and health concerns [13]. These alternations can make neurobehavioral and respiratory disturbances and disrupt circadian rhythms and melatonin secretion, which are related to sleep problems [17–19]. Original studies have investigated the adverse impact of both environmental (indoor and ambient) exposures and occupational exposures to VOCs on sleep issues. However, previous systematic reviews have only investigated the relationships between environmental exposure to air pollutants including VOCs, and sleep [20]. While occupational exposure to VOCs is common in many industries, evidence for the impact of such exposure on sleep health has not been synthesized. The purpose of this study, as a systematic review and meta-analysis, is to summarize the evidence on the effects of occupational exposure to VOCs on sleep quality.

## Methods

This systematic review and meta-analysis study has been registered on PROSPERO (CRD42023391106) and has been written based on the Preferred Reporting Items for Systematic Reviews and Meta-Analyses (PRISMA) guidelines [21]. Furthermore, the PECO framework, for Population, Exposure, Comparator, and Outcome, has been provided to represent a clearer depiction of the relationship between occupational exposure to VOCs and sleep problems [22]. In this systematic review and meta-analysis study, the PECO framework informed the formulation of the research question, as well as the search strategy and criteria for inclusion and exclusion.

### Search Strategy

A systematic search was conducted in five electronic bibliographic databases (Scopus, PubMed, Web of Science, Embase, and Medline) up to 04 March 2024. Three groups of keywords related to occupational exposure to VOCs and sleep problems were used in combination for the search algorithm. The search was conducted without time limitations. The first group of keywords consisted of sleepiness OR sleep OR dyssomnia OR circadian OR parasomnia OR hypersomnia OR Insomnia OR RLS OR restless legs syndrome OR Willis Ekbom Disease OR narcolep OR paroxysmal OR periodic limb movement disorder OR gelineau Syndrome OR nightmare OR somnolence OR nocturnal myoclonus syndrome. The second set of keywords comprised gas OR Vapor OR solvent OR vapour OR VOC OR volatile organic compound. The third set of keywords included industry OR occupation OR industrial OR employee OR workplace OR worker OR workforce.

### Inclusion and Exclusion Criteria

This systematic review and meta-analysis study included studies that investigated associations between occupational exposure to VOCs and sleep problems. Studies addressing all types of sleep problems and study designs were included without any time restrictions. Review articles, meta-analyses, conference papers, editorial letters, case reports, and trial studies were excluded from the study. Animal studies were also not included.

### Study Selection

To select relevant studies, all identified papers were imported into Endnote and duplicates were omitted. Then, two independent reviewers (S.Y. and A.KH) assessed the papers for eligibility and chose the relevant studies. The remaining studies underwent a thorough review to confirm the criteria.

### Quality Assessment

The quality of experimental, cohort, cross-sectional, and case-control studies was assessed using the critical appraisal tools provided by the Joanna Briggs Institute (JBI) [23]. The number of positive responses obtained from each checklist was summed to categorize articles into three groups: low quality, moderate quality, and high quality.

### Risk of Bias Assessment

The potential for bias within the chosen studies was assessed using the Risk of Bias in Non-randomized Studies of Exposures (ROBINS-E) instrument by two independent evaluators, S.Y. and A.KH. This process involved an examination of seven distinct factors: confounding variables, selection bias, deviations from intended exposures, accuracy of exposure measurement, outcome measurement, missing data, and the clarity of reported findings. Each study underwent a separate risk of bias (ROB) analysis, with outcomes categorized as “low,” “moderate,” or “high” in terms of bias risk. An overall classification of “high” bias risk was assigned if any single criterion received a “high” rating. Conversely, a study was deemed to have a “low” overall bias risk if all criteria were marked as “low” risk. In cases where these conditions were not met, the study was designated as having a “moderate” overall risk of bias.

### Data Extraction

Following the selection of relevant studies, information extraction was performed by the two reviewers. This information consisted of details such as the author’s name, publication year, origin country, sample size, job type, age range, gender distribution, study type, work experience, sources of pollutants, concentration levels of pollutants, types of pollutants, duration of pollutant exposure, sleep-related information, prevalence rates of sleep problems or disorders, tools used for assessment purposes, and outcomes observed in the studies.

### Data Analysis

To determine the agreement between the reviewers in the data extraction process, Cohen’s kappa coefficient was utilized [24]. The obtained kappa coefficients for the first and second steps were 0.91 and 0.94 respectively. In this study, the pooled values of odds ratios and prevalence related to sleep problems due to exposure to VOCs were calculated by meta-analysis. To assess heterogeneity in occupational exposure to VOCs, the Cochrane Q-test was applied [25]. Additionally, I^2^ was computed as the proportion of variability due to heterogeneity [26]. For subgroup analyses, nations were grouped based on economic status from low and middle-income (LMIC) to high-income (HIC) following World Bank classifications [27]. Furthermore, the studies were categorized into five global regions: Europe, East/Southeast Asia/Oceania, the Middle East, North America, and Africa. Based on temporal data, studies were divided into those performed in or before 2010 and after 2010. Data was analyzed by STATA version 14.2.

## Results

### Search Results and Study Selection

In this systematic review and meta-analysis, a total of 4,097 papers were identified across various databases up to March 4, 2024. From these papers, 2,199 redundant studies were excluded. Subsequently, two investigators meticulously reviewed the titles and abstracts of the remaining 1,898 papers. Through this rigorous screening, an additional 1,861 papers were deemed irrelevant as those did not satisfy the inclusion criteria. Ultimately, 37 papers were entered into the present study [28].

### Specification of the Articles

Table 1 presents the specifications of the articles that have been included in this systematic review. Out of the 37 research, 16 research were categorized as cross-sectional studies, 18 research were classified as case-control studies, two research were experimental studies, and one research was a cohort study. Of the studies, two were from South Korea, two from France, one from Australia, two from the United States, one from India, one from Singapore, one from Venezuela, four from Finland, four from Norway, two from Japan, two from Belgium, one from New Zealand, three from Sweden, one from Thailand, three from Turkey, two from Germany, one from Algeria, one from Hungary, one from Czechoslovakia, one from Poland, and one from Malaysia.

**TABLE 1:** The specification of the articles included in this systematic review - Iran, 2024.

First author (Year)	Country	Sample size	Study type	Job type	Range/mean age (years) and gender	Work experience (years)	Pollutant sources	Concentration	Type of pollutant	Pollutant exposure duration (hr)	Sleep Information (prevalence)	Tool of sleep quality	Outcome	Quality
Cho and Kang (2022) [29]	South Korea	36996	Cohort	General working population	15 to ≥60 Male and female	-	-	-	Vapors of solvent	-	Sleep disturbance (6.1%)	Minimal Insomnia Symptom Scale (MISS)	Exposure to vapors of solvent was positively associated with sleep disturbance (OR = 4.1)	Q1
Lucas et al. (2015) [30]	France	145	Case-control	Dry-Cleaning Employees	42 Male-female	3	dry-cleaning operations	7 (ppm, air) and 73.6 (μg/l, blood)	Perchloroethylene	6 to 10	Sleepiness	Epworth’s validated scale	The exposed population did not exhibit an abnormal amount of drowsiness	Q2
Jay et al. (2017) [31]	Australia	2,961	Cross-sectional	General working population	20 to ≥55 Male and female	-	Combustion products and engine emission	-	Vapors of solvents	≥8	Sleepiness (People with standard hours: 5.6% and With non-standard hours: 9.6%)	Epworth Sleepiness Scale (ESS)	The workers with non-standard hours are more likely to be at a higher risk of being exposed to workplace hazards and experiencing insufficient sleep and excessive sleepiness	Q2
Gallicchio et al. (2011) [32]	United States	961	Cross-sectional	Cosmetologist	42.95 Female	-	Cosmetology activities	-	Vapors of solvents	-	Sleep disturbance (33.1%)	Sleep survey	Handling cleaning supplies was found to have a significant correlation with frequent disturbances in sleep (OR = 1.64)	Q1
Tripathi et al. (1989) [33]	India	95	Case-control	Painters	32.75 Male	10.63	High-pressure spray painting	0.59 to 4.63 (mg/m^3^, air)	Volatile solvents such as mineral spirit, xylene, and thinner	4	Sleepiness	Visual analogue scales	Noteworthy negative effects on levels of sleepiness were observed among painters	Q3
Ng et al. (1990) [34]	Singapore	223	Case-control	Printing and paint workers	32.8 Male	9.9	-	-	mixture of organic solvents	-	Sleep disturbance (20.5%)	Structured questionnaire	An excess of symptoms of sleep disturbances was seen in the exposed group.	Q2
Escalona et al. (1995) [35]	Venezuela	149	Case-control	Workers in adhesive factory	31.5 Male and female	7	immersing into vats of solvent mixtures, drying, and cutting rolls	Toluene = 58 ppm; Xylene = 5.1 ppm; Pentane = 5.6 ppm; Heptane = 2.2 ppm; Hexane = 4.6 ppm; n-Hexane = 2.2 ppm; Benzene = 0.3 ppm	Vapors of solvents	-	Insomnia (15%) and wakeful sleep (43%)	Self-report questionnaire	The group that was exposed to the stimulus exhibited a higher occurrence of sleep disruptions in comparison to the group that was not exposed	Q1
Antti-Poika et al. (1982) [36]	Finland	361	cross-Sectional	Painters, workers in paint factories, carpet workers, workers in furniture factories, and laundry workers	38.6 Male and female	10.4	-	-	Trichloroethylene, perchloroethylene and solvent mixtures	-	Sleep disturbances (22%)	Self-report questionnaire	It was found that the exposure can significantly cause sleep disturbances	Q2
Laine et al. (1993) [37]	Finland	21	Experimental	-	19 to 23 Male	-	Experimental chamber	200–400 ppm	m-xylene	-	Poor sleep quality	Static charge sensitive bed (SCSB) device	The effects of exposure to m-xylene at a consistent concentration of 200 ppm on the sleep patterns of subjects were investigated using static charge-sensitive bed recording. It was found that this exposure did not have a significant impact on the ratio of “active” to “quiet” sleep. However, there was a slight decrease in the number of body movements during sleep	Q1
Monstad et al. (1992) [38]	Norway	85	Case-control	House painters, car painters, and garage mechanics	46.8 Male and female	-	-	-	Organic solvents	-	Sleep apnea (35%)	Polysomnography	Individuals who were no longer exposed to solvents exhibited a significantly lower apnea index compared to those who had recently been exposed (P < 0.05)	Q1
Takeuchi et al. (1975) [39]	Japan	4	Cross-sectional	Brocade sashes cleaners	16 to 34 Male and female	-	Solvent	1,250 ppm	Petroleum benzine	8 to ≥12	Insomnia	Self-report questionnaire	The findings suggest that benzene may contribute to insomnia	Q3
Vouriot et al. (2005) [40]	France	43	Case-control	Workers in serigraphy company	36.3 Male and female	6	-	Benzene hydrocarbons = 80.1 mg/m^3^; 1-methoxy-2-propyl acetate = 20.4 mg/m^3^	Aromatic hydrocarbons	8	Poor sleep quality (9%)	Epworth sleepiness scale (ESS) and Pittsburgh sleep quality index (PSQI)	Workers who were exposed to vapors reported no disturbances in sleep quality when compared to control subjects	Q1
Godderis et al. (2011) [41]	Belgium	48	Case-control	Printing plant workers	33.8 Male	13	Cleaners of printing presses	-	Solvents	-	Sleep apnea (67%)	Sleep-related complaints questionnaire and polysomnography	The correlation between the sleep complaint scores and both the exposure index and duration was found to be positive (P = 0.01) in this study. Additionally, the polysomnography results revealed a higher occurrence of central apneas in the workers who were exposed (67%) compared to the referents (30%)	Q1
Kaukiainen et al. (2009) [42]	Finland	368	Cross-sectional	General working population	55.3 Male	10.8	-	-	Solvents	-	Sleep disturbance	Euroquest questionnaire	Sleep disturbance was found to be significantly more prevalent in cases of chronic solvent-induced encephalopathy compared to non-exposed cases (P < 0.001)	Q2
Keer et al. (2016) [43]	New Zealand	581	Case-control	Vehicle collision repair workers	36.5 Male	17	Paint panel beat and spray painters	Panel Beaters = 0.57 ppm; Spray Painters = 2.26 ppm	Propanols, methyl ethyl ketone, benzenes, hexanes, and ethanol	-	Sleep Disturbance (9.5)	Euroquest questionnaire	The employees involved in collision repair exhibited a considerably higher prevalence of sleep disturbance symptoms compared to the reference workers (OR = 1.8)	Q1
Kraut et al. (2015) [44]	United States	19	Cross-sectional	Sewage treatment workers	43 Male	8.1	-	22–52 mg/L, urinary phenol level	Solvents	8	Sleep requirement (26%)	Self-report questionnaire	Fourteen individuals (comprising 74% of the sample) reported experiencing symptoms related to the central nervous system (CNS) that are commonly associated with exposure to solvents. These symptoms include feelings of dizziness, fatigue, an increased need for sleep, and headaches	Q2
Lindelof et al. (2010) [45]	Sweden	211	Case-control	House painters, spray finishers, and printers	52 Male	-	-	-	Organic solvents	-	Insomnia (22%)	Questionnaire	The findings demonstrated a greater occurrence of insomnia within the population exposed to solvents	Q2
Tjalvin et al. (2015) [19]	Norway	284	Case-control	Industrial harbor area workers	44.5 Male and female	-	Chemical explosion		Hydrocarbons	-	Sleep problems	Subjective health complaints inventory	sleep problems were significantly different between exposed employees and controls	Q2
Monstad et al. (1987) [46]	Norway	19 (group 1 = 15; group 2 = 8; control = 3)	Cross-sectional	Garage mechanic, vulcanization, offset printer, house painting, dry cleaning, plastic boat factory, gunsmiths	37.7 Male and female	-	-	-	Acetone, trichloroethane, toluene, and xylol	-	Sleep apnea (46%)	Polysomnography	The findings indicate that the occurrence of sleep apnea can be attributed to exposure to organic solvents	Q1
Edling et al. (1993) [47]	Sweden	66	Cross-sectional	General working population	53 Male	24	-	-	Organic solvents	-	Sleep apnea (19.7%)	Static charge sensitive bed and questionnaire	A high prevalence of sleep apnea was observed among a group of workers who were regularly exposed to organic solvents during their work activities	Q3
Heo et al. (2013) [13]	South Korea	7,112	Cross-sectional	Korean workers	<30 to ≥60 Male and female	<1 to ≥10	-	-	Organic solvents	≥8	Sleep disturbance (28%)	Self-report questionnaire	Exposure to chemical substances has been found to have a substantial correlation with disruptions in sleep patterns (OR = 1.57)	Q2
Thetkathuek et al. (2015) [48]	Thailand	192	Case-control	Workers in paint manufacturing	33.33 Male and female	6.2		Xylene = 2.7 ppm, air; toluene = 9.5 ppm, air; methyl hippuric acid = 78 mg/g, urine	Toluene and xylene	-	Sleep disturbances (46.7%)	Interview Form	The study revealed that individuals exposed to xylene experienced a significant increase in sleep disturbance, with an odds ratio (OR) of 3.9. Similarly, those exposed to toluene showed an even higher likelihood of sleep disturbance, with an OR of 4.4	Q1
Sağcan et al. (2018) [49]	Turkey	32	Case-control	Street sign painters	28.5 Male	7.9	-	-	Toluene and acetone	11	sleep apnea (44%)	Polysomnography and Epworth sleepiness scale (ESS)	A significant number of workers who were regularly exposed to solvents experienced sleep apnea	Q2
Heiskel et al. (2002) [50]	Germany	443	Case-control	-	20 to 89 Male	-	-	-	Paints and solvents	-	Obstructive sleep apnea	Questionnaire	There was no evidence of a relationship between exposure to solvents and the occurrence of obstructive sleep apnea	Q1
Mandiracioglu et al. (2011) [51]	Turkey	263	Cross-sectional	Furniture enterprises workers	15 to ≥50 Male	5 to 24	Painting and varnishing	benzene = 0.11 ppm, blood; toluene = 0.43 ppm, blood	toluene, xylene and benzene	≥8	Sleep disturbances	Euroquest questionnaire	There were no observed differences in the mean scores of the neuropsychological symptoms questionnaire between the groups that were exposed and those that were not exposed	Q2
Levy et al. (1997) [52]	Norway	-	Cross-sectional	Painters and workers in the printing industry	- Male and female	-	-	-	Solvents	-	Sleep Disturbances	Self-report questionnaire	Subjects experienced disruptions in their sleep patterns as a result of their condition known as multiple chemical sensitivity (MCS)	Q2
Laire et al. (1997) [53]	Belgium	42	Case-control	Offset printers	38.1 Male and female	15	-	-	Solvents	-	Nocturnal desaturation	Pulse oximeter	The incidence of nocturnal desaturation was significantly elevated among the workers who were exposed to the hazardous conditions	Q1
Sekkal et al. (2016) [54]	Algeria	500	Case-control	Handling/distributing petroleum products workers	41 Male	14	handling/distributing petroleum products	-	Hydrocarbons	-	Poor sleep quality (15.6%)	Epworth questionnaire and Berlin questionnaire	The prevalence of sleep disturbances was found to be greater among employees who were exposed to the vapors, as compared to those who were not exposed	Q1
Lovas et al. (2021) [55]	Hungary	258	Case-control	Warehouse workers	39.7 Male and female	-	Handling cargos	-	Volatile organic compounds (VOCs)	-	Sleep disturbances	European Health Interview survey	The subjects who were exposed to the vapors experienced a higher occurrence of subjective sleep disturbance symptoms compared to those who were not exposed	Q1
Kaukiainen et al. (2009) [56]	Finland	2,000	Cross-sectional	Painters and carpenters	48.7 Male	11.8	-	-	Solvents	-	Sleep disturbances	Euroquest-based questionnaire	Sleep disturbances were frequently documented during the clinical assessment	Q1
Ulfberg et al. (1997) [57]	Sweden	1,348	Case - control	-	50.6 Male and female	-	-	-	Solvents	-	Obstructive sleep apnea syndrome (OSAS) and snoring	Oximetry and respiratory movement monitoring, static charge sensitive bed (SCSB) and questionnaire	The likelihood of developing Obstructive Sleep Apnea Syndrome (OSAS) or snoring was found to increase in correlation with greater levels of exposure	Q1
Saygun et al. (2012) [58]	Turkey	389	Case-control	Workers in a gun factory	41.4 Male	17.74	-	-	Solvents	8	Daytime sleepiness	Questionnaire	The group exposed to solvents exhibited a greater level of daytime sleepiness	Q2
Kellerova et al. (1985) [59]	Czechoslovakia	394	Cross-Sectional	-	-	-	-	-	Organic solvents	-	Sleep phenomena (37.5%)	Electroencephalography	The rapid onset of deeper sleep stages was commonly associated with benzene exposure	Q3
Indulski et al. (1996) [60]	Poland	418	Cross-Sectional	Workers in paint and varnish production	20 to 59 Male and female	17.34	-	-	Organic solvent mixtures	-	Sleep disorders and sleepiness during the day	Electroencephalography	Sleep disorders and excessive sleepiness were the prevailing concerns reported by the exposed group throughout the daytime	Q3
Kiesswetter et al. (1997) [61]	Germany	-	Experimental	-	-	-	-	200–1,000 ppm	Organic solvents	-	Poor sleep quality	Self-report questionnaire	The employees exposed to solvents experienced a decrease in the quality of their sleep when compared to the control group	Q2
Takeuchi et al. (1972) [62]	Japan	2	Cross-Sectional	paints industry workers	- Male	-	-	-	Toluene	-	Insomnia	Self-report questionnaire	The participants expressed dissatisfaction with their inability to fall asleep and stay asleep	Q3
Syazawani Shamsudin (2023) [63]	Malaysia	42	Cross-Sectional	Office employees	<40 to ≥40 Male and female	<2 to ≥2	-	0.84 ppm	Volatile organic compounds (VOCs)	8	Sleepiness (80.25%)	Self-report questionnaire	The results showed that sleepiness was a symptom of the sick building syndrome because of exposure to pollutants among employees	Q3

NOTE:

^a^ Epworth sleepiness scale (ESS): Scores >10 are considered to be a sign of moderate and severe sleepiness.

^b^ Stop-Bang questionnaire: scores ≥3 are considered to be a sign of moderate and severe risk for OSA.

Among these studies, four research were conducted on the general working population, three on cleaning workers, one on cosmetologists, 15 on painters and workers in paint factories, two on mechanics and car repair workers, and two on furniture factories. Other studied populations included carpet workers, laundry workers, workers in adhesive factories, sewage treatment workers, spray finishers, industrial harbor area workers, handling/distributing petroleum products workers, warehouse workers, workers in gun factories, and office employees.

**FIGURE 1:**
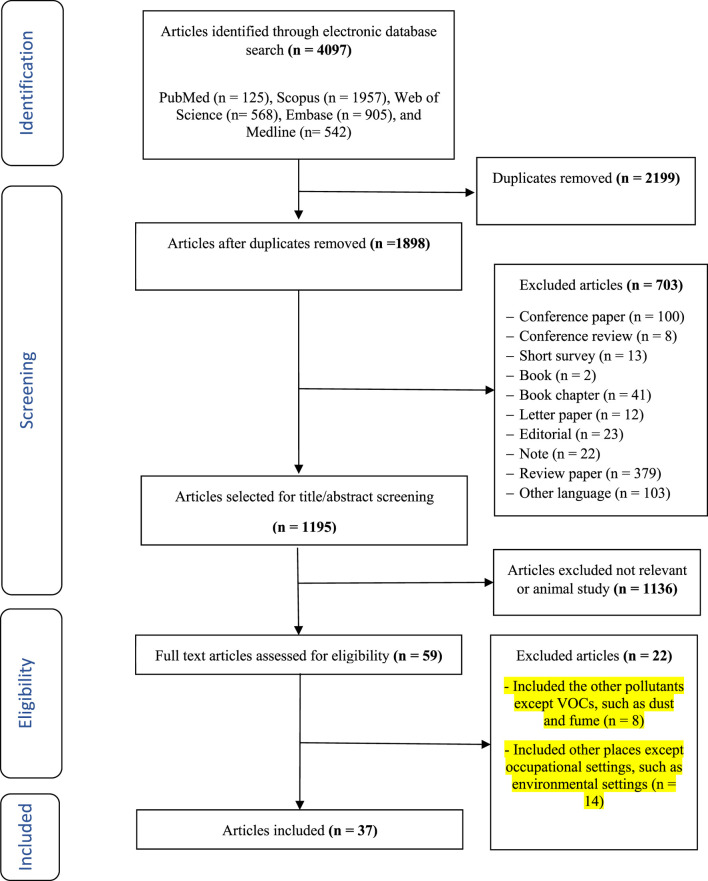
PRISMA (Preferred Reporting Items for Systematic Reviews and Meta-Analyses) flow diagram - Iran, 2024.

Of the studies, 16 were carried out among males, one among females, and 20 among both males and females. Of the studies, 12 investigated sleep disturbance, 6 assessed sleepiness, 4 assessed insomnia, 4 assessed poor sleep quality, 7 assessed sleep apnea, and 4 evaluated other sleep problems. Based on the quality assessment, 16 were categorized as high quality (43.24 percent), 14 as moderate quality (37.84 percent), and 7 as low quality (18.92 percent) (Figure 1).

### Qualitative Findings

**FIGURE 2:**
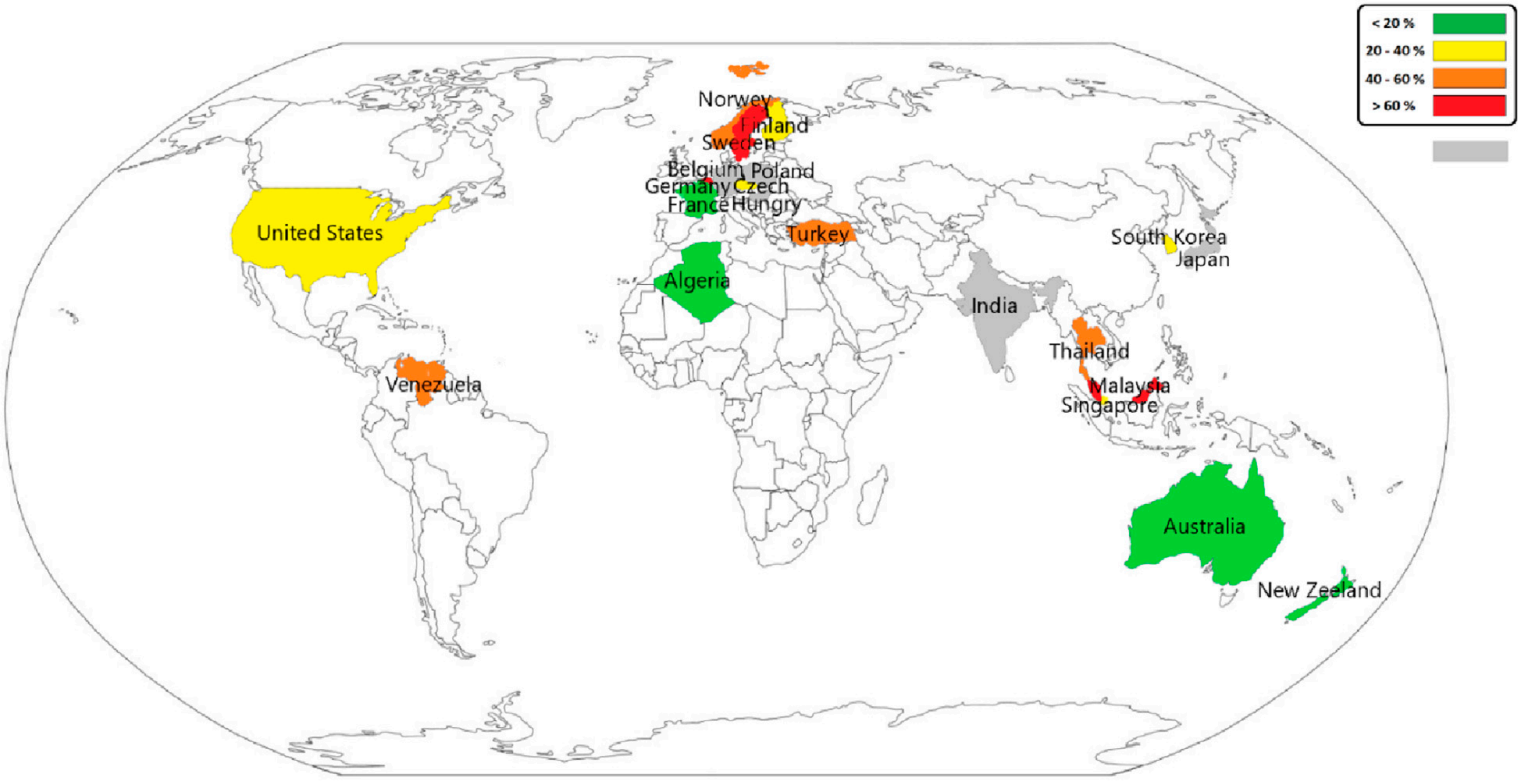
Global map of the prevalence of sleep problems due to occupational exposure to volatile organic compounds - Iran, 2024.

Figure 2 illustrates the global map of the prevalence of sleep problems due to occupational exposure to VOCs. Based on the results, the highest sleep problems due to this exposure were found in Sweden due to exposure to VOCs among the general working population (82.5 percent) and Malaysia due to exposure to VOCs among office employees (80.25 percent), respectively.

Among 16 articles with high quality, 13 (81.25%) showed that exposure to VOC had a significant negative effect on sleep among workers in various occupations. Among 14 articles with moderate quality, 12 studies (85.71%) showed that exposure to VOC had a significant effect on sleep among workers in various occupations. Among 7 articles with low quality, all (100.00%) indicated that exposure to VOC had a significant effect on sleep among workers in various occupations.

**FIGURE 3:**
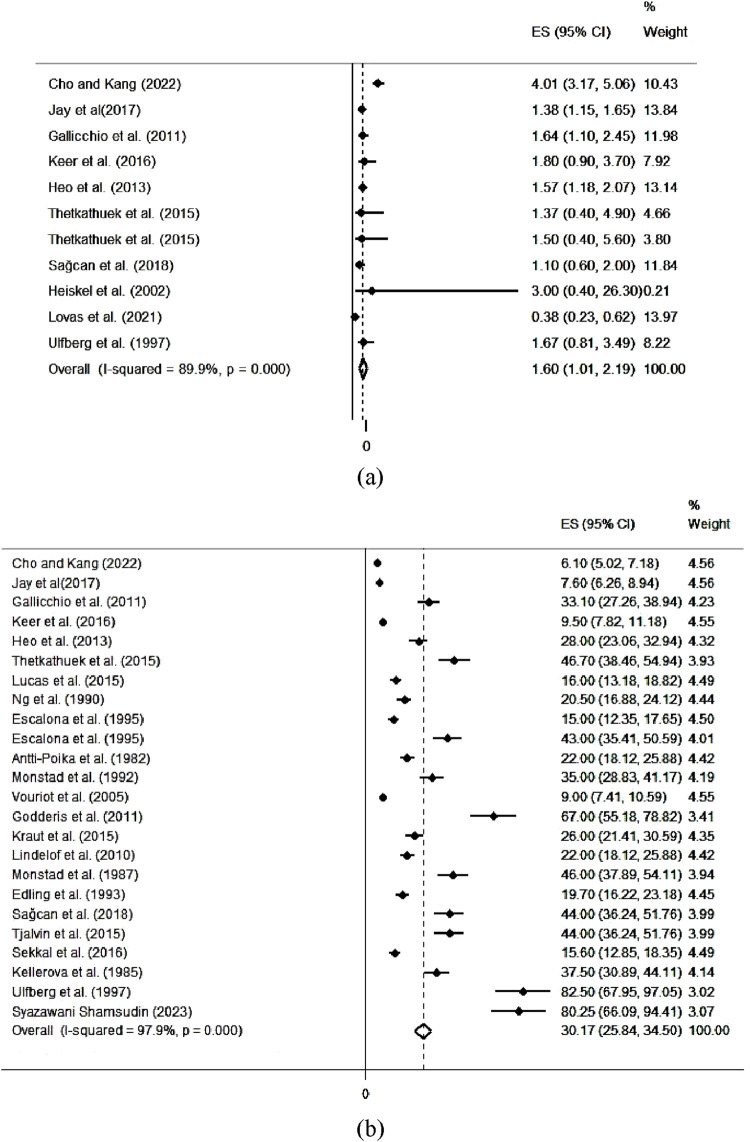
The pooled values of outcomes related to sleep problems due to occupational exposure to volatile organic compounds. **(a)** odds ratio and **(b)** prevalence - Iran, 2024.

Figure 3; Table 2 show the pooled values of outcomes related to sleep problems due to exposure to VOCs. The results of the meta-analysis revealed that the pooled value of the odds ratio in the studies was 1.60 [95% CI (1.01, 2.19)]. Also, the results of the meta-analysis indicated that the pooled value of the prevalence in the studies was 30.17 [95% CI (25.84, 34.50)]. Moreover, Table 3 completely describes the findings of the subgroup analysis for the odd ratios and prevalences. The pooled values of odds ratios were significantly greater in countries with high incomes {1.70 [95% CI (1.01, 2.40)]} compared to those with low and medium incomes, in the East/Southeast Asia/Oceania {2.00 [95% CI (1.24, 2.77)]} compared to other regions, and in 2010 or earlier {1.68 [95% CI (0.35, 3.02)]} compared to after this time. The pooled values of prevalence were significantly higher in countries with low and medium incomes {39.48 [95% CI (26.49, 52.46)]} compared to those with high incomes, in the Middle East {44.00 [95% CI (36.24, 51.76)]} compared to other regions, and in 2010 or earlier {33.96 [95% CI (26.58, 41.34)]} compared to after this time.

**TABLE 2:** The results of meta-analysis on outcomes related to sleep problems due to occupational exposure to volatile organic compounds - Iran, 2024.

Outcome type	Authors (year)	Effect size	95% confidence intervals	Weight (%)
Odds ratio	Cho and Kang [29]	4.01	(3.17–5.06)	10.43
	Jay et al. [31]	1.38	(1.15–1.65)	13.84
	Gallicchio et al. [32]	1.64	(1.10–2.45)	11.98
	Keer et al. [43]	1.80	(0.90–3.70)	7.92
	Heo et al. [13]	1.57	(1.18–2.07)	13.14
	Thetkathuek et al. [48]	1.37	(0.40–4.90)	4.66
	Thetkathuek et al. [48]	1.50	(0.40–5.60)	3.80
	Sagcan et al. (2018) [49]	1.10	(0.60–2.00)	11.84
	Heiskel et al. [50]	3.00	(0.40–26.30)	0.21
	Lovas et al. [55]	0.38	(0.23–0.62)	13.97
	Ulfberg et al. [57]	1.67	(0.81–3.49)	8.22
	Overall	1.60	(1.01–2.19)	100.00
prevalence	Cho and Kang [29]	6.10	(5.02–7.18)	4.56
	Jay et al. [31]	7.60	(6.26–8.94)	4.56
	Gallicchio et al. [32]	33.10	(27.26–38.94)	4.23
	Keer et al. [43]	9.50	(7.82–11.18)	4.55
	Heo et al. [13]	28.00	(23.06–32.94)	4.32
	Thetkathuek et al. [48]	46.70	(38.46–54.94)	3.93
	Lucas et al. [30]	16.00	(13.18–18.82)	4.49
	Ng et al. [34]	20.50	(16.88–24.12)	4.44
	Escalona et al. [35]	15.00	(12.35–17.65)	4.50
	Escalona et al. [35]	43.00	(35.41–50.59)	4.01
	Antti-Poika et al. [36]	22.00	(18.12–25.88)	4.42
	Monstad et al. [38]	35.00	(28.83–41.17)	4.19
	Vouriot et al. [40]	9.00	(7.41–10.59)	4.55
	Godderis et al. [41]	67.00	(55.18–78.82)	3.41
	Kraut et al. [44]	26.00	(21.41–30.59)	4.35
	Lindelof et al. [45]	22.00	(18.12–25.88)	4.42
	Monstad et al. (1987) [46]	46.00	(37.89–54.11)	3.94
	Edling et al. [47]	19.70	(16.22–23.18)	4.45
	Sagcan et al. (2018) [49]	44.00	(36.24–51.76)	3.99
	Tjalvin et al. [19]	44.00	(36.24–51.76)	3.99
	Sekkal et al. [54]	15.60	(12.85–18.35)	4.49
	Kellerova et al. [59]	37.50	(30.89–44.11)	4.14
	Ulfberg et al. [57]	82.50	(67.95–97.05)	3.02
	Syazawani Shamsudin [63]	80.25	(66.09–94.41)	3.07
	Overall	30.17	(25.84–34.50)	100

**TABLE 3:** The results of subgroup analysis for the odd ratios and prevalence related to sleep problems due to occupational exposure to volatile organic compounds - Iran, 2024.

Subgroup analysis	Subgroup	Category (number of studies)	Pooled prevalence (%) [95% CI]	*I* ^2^ (%)	*Q* statistic (*df*)	*p* of heterogeneity
Odds ratio	Income level	High income (8) LMICs (3)	1.70 [1.00, 2.40] 1.14 [0.50, 1.79]	92.9 0.0	7 2	<0.0001 0.93
	Region	Europe (3) East/southeast Asia/Oceania (6) Middle East (1) North America (1) Africa (−)	0.80 [-0.21, 1.82] 2.00 [1.23, 2.76] 1.10 [0.40, 1.80] 1.64 [0.96, 2.31] -	45.1 82.1 - - -	2 5 0 0 -	0.16 <0.0001 - - -
	Study date	In or before 2010 (2) After 2010 (9)	1.68 [0.35, 3.01] 1.59 [0.96, 2.22]	0.0 91.8	1 8	0.84 <0.0001
prevalence	Income level	High income (18) LMICs (6)	27.39 [22.79, 31.99] 39.47 [26.49, 52.45]	97.9 97.6	17 5	<0.0001 <0.0001
	Region	Europe (11) East/southeast Asia/Oceania (7) Middle East (1) North America (4) Africa (1)	34.84 [26.64, 43.05] 23.93 [17.79, 30.08] 44.00 [36.23, 51.76] 28.93 [17.30, 40.56] 15.60 [12.84, 18.35]	97.6 98.0 - 95.9 -	10 8 0 3 0	<0.0001 <0.0001 - <0.0001 -
	Study date	In or before 2010 (12) After 2010 (12)	33.95 [26.57, 41.34] 26.96 [21.42, 32.50]	97.5 98.1	11 11	<0.0001 <0.0001

### Assessment of Risk of Bias in Studies

The evaluation results of the seven ROB criteria for each of the articles have been reported in Supplementary Table S1. Out of the 37 selected articles, 67.6% (N = 25) had a high ROB rating, 21.6% (N = 8) were rated as moderate, and 10.8% (N = 4) had a low ROB. The highest percentage of high ROB was observed in the criteria of measurement of exposure and departure from exposure with 67.6% (N = 25). Also, selection bias was observed at a low level in 89.2% of studies.

## Discussion

To the best of our knowledge, this is the first systematic review and meta-analysis study to investigate the associations between volatile organic compounds and sleep quality. In 81.25% of the high-quality studies (13 out of 16 studies), 85.71% of the moderate-quality studies, (12 out of 14 studies), and all of the low-quality studies (7 out of 7 studies), there was a consistent finding that exposure to volatile organic compounds (VOCs) adversely affected sleep in workers across various professions. It has been proposed that this exposure can affect sleep in three different ways, including neurobehavioral, chronic illnesses, and psychological.

One potential mechanism that can explain the impact of daytime occupational volatile organic compounds (VOC) exposure on sleep is the neurobehavioral effect resulting from chemical exposure. Exposure to VOC mist or vapors can directly or indirectly affect the nervous system, leading to neurobehavioral effects such as sleep problems [12]. This may be attributed to autonomic nervous dysfunction [34]. Extensive documentation exists regarding the neurotoxic effects of solvents, including VOCs, which can have toxic effects on the peripheral and central nervous system (CNS) [35]. Prolonged exposure to aromatic hydrocarbons is well-known to be associated with disruptions in CNS functioning [40]. The specific effects depend on the chemical composition of the VOCs [40]. Numerous studies have investigated this relationship [33, 41–43, 56].

As a second pathway, volatile organic compounds (VOCs) exposure can cause chronic illnesses. These include local inflammation of the airways, difficulty breathing leading to chronic bronchitis, and sleep apnea, which disrupts an individual’s comfort and results in sleep problems [14]. Prolonged exposure to solvents can specifically cause sleep disturbances, particularly sleep apnea syndromes [64]. Exposure to solvents may be associated with the occurrence of obstructive sleep apnea syndrome [40]. Additionally, certain VOCs may be associated with allergic reactions that can also cause sleep apnea [65]. Symptoms resulting from allergic and nonallergic rhinitis are considered agents affecting sleep apnea [66]. Potential mechanisms for this association consisted of the impact of nasal obstruction on the collapsibility of the nasopharynx downstream and inflammation related to increased nasal resistance [67]. Furthermore, exposure to certain VOCs can influence the central nervous system and consequently lead to central sleep apnea [38]. Previous studies have reported the potential impact of inhalational occupational exposures to various VOCs on sleep apnea [38, 41, 46, 47, 49]. Moreover, studies have shown that exposure to solvents such as acetone, methacrylate, and acetonitrile can result in asthma, eye irritation, skin irritation, nasal irritation, throat irritation, and dermatitis, which those can affect sleep quality among exposed people [15, 16].

In addition to the mentioned pathways, exposure to volatile organic compounds (VOCs) can induce stress and anxiety due to the annoyance and health concerns associated with direct exposure to hazardous VOCs in unfavorable working conditions [13]. These stressful conditions can increase stress reactions and activate the hypothalamic-pituitary-adrenal axis and rumination [17]. Stress-induced adrenal secretory activity disruption can disturb the rhythmic release of adrenal cortisol [17]. Like other workplace irritants, occupational exposure to VOCs may also contribute to mental disorders [17]. This effect of VOC exposure on mental health can be stated as “VOC annoyance.” A neurocognitive mechanism can explain this concept of mental health. Predisposing factors such as workplace stressors relate to perpetuating factors like extended time spent in bed due to obstacles in achieving de-arousal caused by cortical arousal [68]. These alterations in the human body can lead to sleepiness and sleep problems. Previous studies have shown that chronic stress has a relationship with sleep problems. Findings from some previous studies propose a mechanism mediated by smell perception and smell annoyance for the health effects resulting from solvent exposure [69–71]. Additionally, concerns about potential health risks have been reported to increase subjective health complaints [18, 19].

The findings of the meta-analysis revealed that the pooled value of the odds ratio related to sleep problems due to exposure to VOCs in the studies was equal to 1.60. On the other harmful factors in occupational settings, Heo et al. determined that physical agents could be associated with sleep disruptions, exhibiting an odds ratio of 1.47. In this research, they found that psychosocial factors might influence the sleep quality of employees, with odds ratios ranging from 1.45 to 2.93 [13]. Virtanen et al. discovered that prolonged work durations might correlate with a higher likelihood of reduced sleep duration and increased early morning awakenings, with odds ratios of 3.24 and 2.23, respectively [72]. Heiskel et al. concluded that solvent exposure could contribute to obstructive sleep apnea, with an odds ratio of 1.2 [50]. These results collectively suggest that chemical solvents, the same as other job hazards, may have a significant association with sleep-related issues.

The findings of the meta-analysis indicated that the pooled value of the prevalence of sleep problems due to exposure to VOCs in the studies was 30.17. The findings of previous research have shown that sleep disturbances among employees occur at a rate of nearly 18 percent in European countries and 23 percent in the United States [73, 74]. Cho et al. noted that sleep disturbances due to physical or chemical exposures had a prevalence between 10 and 26 percent [29]. Bertrais et al. observed that occupational hazards were associated with an increased occurrence of sleep problems (30 percent) in male workers [75]. The prevalence of sleep problems obtained in the current study is consistent with previous studies.

The results of subgroup analysis showed that the pooled values of odds ratios were significantly higher in countries with high incomes, in East/Southeast Asia/Oceania, and in 2010 or earlier. While on the prevalence of sleep problems, the pooled values were higher in countries with low and medium incomes, in the Middle East, and in 2010 or earlier. In nations with lower economic status, outdated industrial equipment often operates at reduced energy efficiency levels, leading to elevated pollutant emissions. Additionally, these countries may depend on less efficient energy sources like coal and mazut, contributing to health issues associated with air pollution. The lack of effective enforcement of environmental regulations, because of the prohibitive costs associated with pollution control, further increases the risk of pollutant exposure [76]. However, it must be stated that high-income and developed countries are not exempt from contributing to high levels of pollutants, which could be attributed to their more intensive industrial operations [77]. These causes might somewhat describe the findings of the present studies.

In this study, 16 articles were classified as high quality, 14 studies as moderate quality, and 7 studies as low-quality studies. Therefore, these results show that a low number of low-quality studies were included in this systematic review and meta-analysis study. However, the results of the bias assessment revealed that 67.6% of the studies had a high ROB rating, 21.6% of the studies were rated as moderate, and 10.8% of the studies had a low ROB. The low number of studies characterized by a low risk of bias (ROB) might be due to different factors. These include dependence on self-reporting tools or questionnaires to collect data [78–80], lack of detailed information on pollutant concentrations and sleep problems [81–83], failure to disclose exposure status [81, 84], use of same methodologies [85, 86], and instances of incomplete results [87–89].

### Strengths

As a strength, a comprehensive systematic search was conducted across five electronic databases (Scopus, PubMed, Web of Science, Embase, Medline) with no time restrictions, using three groups of keywords covering sleep problems, VOCs, and occupational contexts. This minimized the risk of missing relevant studies. Moreover, duplicates were removed, and two independent reviewers assessed study eligibility, reducing selection bias.

### Limitations

Based on the results, only one cohort study was performed among 37 studies. The prospective type of study design allows greater confidence in examining exposure values to VOCs compared to retrospective studies with the associated problems of recall fallacy. This limitation restricts the ability to draw causal inferences because cross-sectional and case control designs are not examined the temporal sequence between exposure and outcome. In cross-sectional design, exposure and outcome are simultaneously measured. In case-control studies also, previous exposures and outcomes are investigated. So, it is not clear which one happened first. Furthermore, the impact of confounding factors (such as other harmful agents in the workplace) has not been considered in some of the studies, which may affect the effect of exposure to VOCs. Moreover, most of the reviewed studies had no detailed information on pollutant concentrations and sleep problem metrics.

### Conclusion

Based on the results of this systematic review, 32 out of 37 studies (86.49%) showed that occupational exposure to VOCs can significantly influence sleep among workers in various occupations. This exposure can cause a variety of sleep problems. There are three pathways for this impact, including the neurobehavioral effect due to exposure to VOCs on nighttime sleep, the effect of chronic illnesses due to exposure to VOCs on nighttime sleep, and the psychological effects due to exposure to VOCs on nighttime sleep. The results of this study can be helpful to plan the decrease of exposure to VOCs, such as decreased use of VOCs, design of general and local ventilations, and use of personal protective equipment, for preventing sleep disorders in the workplace. Also, people with sleep disorders should be prevented from employment in workplaces with high exposure to VOCs.
