# Supplementary material for: Associations Between Occupational Exposures to Volatile Organic Compounds (VOCs) and Sleep Problems
Source: Public Health Rev. 2025 Sep 25;46:1608224. doi: 10.3389/phrs.2025.1608224 (PMC12507706; doi:10.3389/phrs.2025.1608224)
Supplement: Supplementary file 1 [file DataSheet1.pdf]

Table S1. Evaluation of the risk of bias in the studies

First Author	Confounding	Selection bias	Departure from exposure	Measurement of exposure	Measurement of outcomes	Missing data	Reported results	Study level
Cho and Kang (2022) [20]	🙂	🙂	🙁	🙁	🙂	😐	😐	🙁
Lucas et al. (2015) [21]	🙂	🙂	🙂	🙂	🙂	🙂	🙂	🙂
Jay et al. (2017) [22]	🙂	🙂	🙁	🙁	🙂	😐	😐	🙁
Gallicchio et al. (2011) [23]	🙂	🙂	🙁	🙁	🙂	🙂	😐	🙁
Tripathi et al. (1989) [24]	🙂	🙂	🙂	🙂	🙂	🙂	🙂	🙂
Ng et al. (1990) [25]	🙂	🙂	🙁	🙁	🙂	😐	😐	🙁
Escalona et al. (1995) [26]	😐	🙂	🙂	🙂	😐	😐	😐	😐
Antti-Poika et al. (1982) [27]	🙁	🙂	🙁	🙁	🙁	🙂	😐	🙁
Laine et al. (1993) [28]	🙂	🙂	🙂	🙂	🙂	🙂	🙂	🙂
Monstad et al. (1992) [29]	🙂	🙂	🙁	🙁	🙂	😐	😐	🙁
Takeuchi et al. (1975) [30]	😐	🙂	🙂	🙂	😐	🙂	🙂	😐
Vouriot et al. (2005) [31]	🙂	🙂	🙂	🙂	🙂	🙂	🙂	🙂
Godderis et al. (2011) [32]	😐	🙂	🙁	🙁	🙂	🙂	😐	🙁
Kaukiainen et al. (2009) [33]	🙁	🙂	🙁	🙁	🙁	😐	😐	🙁
Keer et al. (2016) [34]	😐	🙂	🙂	🙂	😐	🙂	😐	😐
Kraut et al. (2015) [35]	😐	🙂	🙂	🙂	😐	😐	🙂	😐
Lindelof et al. (2010) [36]	🙁	🙂	🙁	🙁	🙁	🙂	😐	🙁
Tjalvin et al. (2015) [37]	🙁	🙂	🙁	🙁	🙁	😐	😐	🙁
Monstad et al. (1987) [38]	😐	🙂	🙁	🙁	🙁	😐	🙂	🙁

First Author	Confounding	Selection bias	Departure from exposure	Measurement of exposure	Measurement of outcomes	Missing data	Reported results	Study level
Edling et al. (1993) [39]								
Heo et al. (2013) [40]								
Thetkathuek et al. (2015) [41]								
Sağcan et al. (2018) [42]								
Heiskel et al. (2002) [43]								
Mandiracioglu et al. (2011) [44]								
Levy et al. (1997) [45]								
Laire et al. (1997) [46]								
Sekkal et al. (2016) [47]								
Lovas et al. (2021) [48]								
Kaukiainen et al. (2009) [49]								
Ulfberg et al. (1997) [50]								
Saygun et al. (2012) [51]								
Kellerova et al. (1985) [52]								
Indulski et al. (1996) [53]								
Kiesswetter et al. (1997) [54]								
Takeuchi et al. (1972) [55]								
Syazawani Shamsudin (2023) [56]								
